# Hospital Finance—A Disclaimer

**Published:** 1888-09-01

**Authors:** Sidney M. Quennell

**Affiliations:** Sec. Westminster Hospital, Broad Sanctuary, S.W.


### THE EDITOR'S LETTER-BOX.

[Our Correspondents are reminded that prolixity is a great bar to publication, and that brevity of style and conciseness of statement greatly facilitate early insertion.]

### HOSPITAL FINANCE—A DISCLAIMER.

MY attention has been called to an article in your issue of this date on "Hospital Finance and Economy," which professes to give the cost per head per annum of the in-patients in various hospitals. I desire to point out that, as regards this hospital, the writer of the article in question has fallen into an error in stating the cost as £89 per head per annum. The calculation is said to have been made from figures supplied for the information of the Council of the Hospital Sunday Fund, and the principles adopted in dealing with those figures is explained as follows:—

"The cost per head has been computed from the gross average expenditure of each institution during the last three years, excluding investments of legacies, repayment of mortgages, cost of new buildings, etc., and is divided by the mean number of patients resident in the hospital during the past year. It would no doubt have been better to have had the average number of resident patients for the three years instead of for one year only."

On referring to the returns which I made this year to the Hospital Sunday Fund, I find that the gross expenditure of this hospital during the three years 1885-6-7 was £57,371 8s. 11d. From this must be deducted the following items, according to the view taken by the writer of the article (*see table below.*)

But I agree in the view expressed by your correspondent, that it would have been better to have had the average number of resident patients for the three years instead of for one year only. The average number of resident patients at this hospital for the three years in question was exactly 160, and if this number be taken as the basis of the calculation, the average cost per annum per head is £70 9*s.* 7*d*., or over 20 per cent. less than the amount which it is said to have been. Your correspondent confesses to having been baffled in his attempt to analyse the figures contained in a miscellaneous collection of hospital reports. I fear he has not been more successful in dealing with the statistics issued by the Council of the Hospital Sunday Fund.

| | | | | | | |
|---|---|---|---|---|---|---|
| New buildings, not provided for by a special fund ...... | £11,459 | 17 | 1 | | | |
| Furniture for new buildings .. | 641 | 7 | 0 | | | |
| *Building medical schools...... | 9,218 | 8 | 8 | | | |
| *Building chapel.................. | 1,421 | 14 | 11 | | | |
| Invested............................. | 800 | 0 | 0 | | | |
| | | | | £23,541 | 7 | 8 |
| Leaving .............................................. | | | | £33,830 | 1 | 3 |
| Dividing this by three, gives an average annual expenditure of ........................................ | | | | £11,276 | 13 | 9 |
| And this again, divided by 172, the average number of in-patients last year, as quoted by the writer of the article, the average cost per head per annum is shown to be............ instead of £89. | | | | £65 | 11 | 3 |

* Provided for in whole or part by special funds.

SIDNEY M. QUENNELL, Sec.

Westminster Hospital, Broad Sanctuary, S.W.
August 25th, 1888.

